# Risk factors for predicting extended-spectrum β-lactamase-producing Enterobacterales (ESBLE) infections in non-urinary isolates

**DOI:** 10.1017/ash.2023.201

**Published:** 2023-07-11

**Authors:** Rachel Burgoon, Aaron Hamby, Erin Weeda, Brian R. Raux, Krutika M. Hornback

**Affiliations:** 1 Department of Pharmacy, Medical University of South Carolina (MUSC) Health, Charleston, SC, USA; 2 Department of Clinical Pharmacy & Outcome Sciences, Medical University of South Carolina, Charleston, SC, USA; 3 bioMerieux, Inc., Salt Lake City, UT, USA

## Abstract

**Background:**

With increases in antimicrobial resistance, it is crucial that patients receive appropriate antimicrobial therapy in a timely manner. Advancements in rapid diagnostics offer the ability to identify resistant organisms quickly. However, this technology is not always accessible and relies on correct specimen collection. While awaiting new microbiology methods, it may be beneficial to identify risk factors associated with common types of resistance. Specifically, extended-spectrum β-lactamase-producing Enterobacterales (ESBLE) are a rising threat globally.

**Objective:**

The primary objective of this retrospective case–control analysis was to identify factors associated with non-urinary ESBLE versus non-ESBLE infections.

**Design/Methods:**

Patient cultures were randomly selected based on type of culture (blood, bacterial, or exudate) and organism (*E. coli*, *K. pneumoniae*, or *K. oxytoca*) to provide a 1:1 ratio of ESBLE to non-ESBLE infections. Baseline demographics and potential risk factors (malignancy, cirrhosis, acute kidney injury (AKI), and diabetes) were collected for each patient encounter.

**Results:**

In the univariate analysis, risk factors that achieved a significant difference included cirrhosis, AKI, presence of urinary catheter, presence of center venous catheter, history of an ESBLE infection, hospital-acquired infection, and recent fluoroquinolone, cephalosporin, or beta-lactam use. The multivariate analysis showed that four factors were independently associated with an ESBLE infection: cirrhosis, urinary catheter, central venous catheter, and history of ESBLE. Having a history of an ESBLE had the highest adjusted odds ratio (aOR 12.49; 95% CI 4.71–33.15, *P* < .001) of the four factors.

**Conclusions:**

These results demonstrate that there may be benefit in incorporating select risk factors into clinical decision support tools to identify patients at highest risk of ESBLE infection.

## Background

Extended-spectrum β-lactamase-producing Enterobacterales (ESBLE) infections pose a serious threat to antibiotic resistance in the United States according to the Centers for Disease Control and Prevention (CDC) *Antibiotic Resistance Threats* report.^1^ Since 2012, the number of cases of ESBLE infections in hospitalized patients has continued to rise. The latest data estimate 197,500 cases of ESBLE infections in 2020 compared to 131,900 cases in 2012.^1^ While a variety of organisms have the ability to express ESBL resistance, the most commonly associated organisms include *Escherichia coli, Klebsiella pneumoniae, Klebsiella oxytoca,* and *Proteus mirabilis*.^2,3^ As this threat increases, identifying potential ESBLE infections and providing proper treatment are critical.

The Infectious Diseases Society of America (IDSA) provides recommendations on definitive treatment of ESBLE infections; however, empiric therapy is key in this population.^2^ This is because organisms can harbor multiple mechanisms of resistance, which increases the risk in our patients.^2^ As carbapenems are the drugs of choice for ESBLE infections, it is important to understand their potential impact. Broad therapy could possibly lead to future ESBLE infections, while overly narrow therapy may not cover a potential ESBLE organism.^3–6^ Additionally, recent studies propose that increased carbapenem use may potentiate carbapenem resistance in organisms like *Klebsiella pneumonia*, which may lead to further difficulties in treating these infections.^5,6^

Previous literature has been published validating risk factor prediction scores for ESBLE infections in hospitalized patients.^3,7–9^ Three studies solely evaluated bloodstream infections, while a study in Italy looked at all available types of cultures.^2,3,8^ Our study strives to further contribute to current literature by including all non-urine isolate infections. The purpose of this study was to evaluate risk factors associated with non-urinary ESBLE versus non-ESBLE infections.

## Methods

### Design

This retrospective case–control analysis was conducted at the Medical University of South Carolina (MUSC) Health, a large academic medical center. Patients > 18 years of age admitted between July 1, 2014 and June 30, 2021, with a documented ESBLE or non-ESBLE organism treated with antibiotics were included in the study for initial review.

Patients were excluded if they had a positive urine culture for ESBLE, multiple cultures growing the same organism of interest (to avoid duplicates), organisms that could harbor the chromosomal AmpC-mediated gene, and organisms that were carbapenem resistant on proven susceptibilities. In this study, organisms considered to harbor chromosomal AmpC-mediated genes included *Hafnia alvei*, *Enterobacter cloacae*, *Citrobacter freundii*, *Klebsiella aerogenes*, *Yersinia enterocolitica*, and *Serratia* species.^2^ Included patients were randomly selected and matched based on type of culture (blood, bacterial, or exudate) and organism (*E. coli, K. pneumoniae,* or *K. oxytoca*) to provide a 1:1 ratio of ESBLE (cases) to non-ESBLE (controls). Our microbiology lab now defines a bacterial culture as any non-blood and non-fungus culture that is not a wound, abscess, or fluid culture. All wound, abscess, and fluid cultures are classified as exudate cultures. As this was a recent change in our microbiology lab protocols, the total number of “bacterial” cultures identified was expected to be smaller in comparison to cultures coded as “blood” or “exudate”.

### Data collection

The culture data and site of infection were provided via the institutional clinical data warehouse. Once the patients were identified, baseline demographics such as age, sex, and race were collected directly from the electronic health record. Other potential risk factors collected included malignancy, chemotherapy administration, cirrhosis, endoscopy, acute kidney injury (AKI), diabetes, immunocompromised status, outpatient gastrointestinal (GI) procedure, presence of urinary catheter, presence of central venous catheter, history of ESBLE, hospital-acquired infection, hospitalization in the previous 90 days, antibiotics in the previous 90 days, length of stay (LOS), and if the patient was admitted from a long-term care facility. Individual antibiotics were evaluated for utilization and categorized into use of cephalosporins, other beta-lactams, or fluoroquinolones.

### Statistical analysis

The primary objective was to identify factors associated with non-urinary ESBLE versus non-ESBLE infections. Data are presented as counts with proportions for categorical data and medians with interquartile ranges for continuous data. A univariate analysis was conducted comparing continuous and categorical data between ESBLE and non-ESBLE groups using Mann–Whitney U and χ^2^/Fisher’s exact tests. Factors with a *P* value ≤ .2 in the univariate analysis were entered into a multivariable logistic regression model. For the multivariable model, backward elimination was utilized, and *P* values < .05 were considered significant. Multicollinearity was considered in both design and analysis phases. Care was taken to not select variables that would be expected to be correlated with others that were already being measured. When two variables that could present overlap were identified, one variable was selected based on clinical judgment. Goodness of fit was assessed using the Hosmer and Lemeshow test. Data analysis was performed using SPSS version 27.

## Results

A total of 3,051 patients between July 1, 2014 and June 30, 2021, were identified; of which, 177 patients with an ESBLE isolate met inclusion criteria. A total of 177 patients with a non-ESBLE (controls) were matched to the included patients with an ESBLE (cases) based on type of culture (blood, bacterial, or exudate) and organism (*E. coli, K. pneumoniae,* or *K. oxytoca)*.

Baseline characteristics were similar between the two groups (Table 1). The most common ESBLE organisms isolated were *E. coli* (129 isolates) and *K. pneumoniae* (42 isolates). Of the 177 ESBLE isolates, 108 were from the blood, 68 from an exudate culture, and 1 from a bacterial culture.

**Table 1.** Univariate analysis for risk factors associated with ESBLE infections in non-urine isolates. Previous fluoroquinolone and previous cephalosporin use were not included in the final model due to concerns with collinearity (specifically, fluoroquinolone and cephalosporin use in patients with cirrhosis where these antibiotics are used as prophylaxis and treatment of spontaneous bacterial peritonitis)

	ESBLE (n = 177) n (%)	Non-ESBLE (n = 177) n (%)	*P* value
Median age, years [IQR]	61 [46–69]	63 [48–72]	.20
Male	98 (55)	88 (50)	.29
Length of stay, days [IQR]	12 [6–22]	7 [4–15]	< .001
Race			
White	103 (58)	81 (46)	.02
African American	65 (37)	91 (51)	
Other	9 (5)	5 (3)	
Risk factors			
Malignancy	44 (25)	36 (20)	.31
Chemotherapy	21 (12)	17 (10)	.49
Cirrhosis	28 (16)	14 (8)	.02
Endoscopy	15 (9)	11 (6)	.42
Acute kidney injury	93 (53)	73 (41)	.03
Diabetes mellitus	69 (39)	64 (36)	.58
Immunocompromised	39 (22)	31 (18)	.29
Resident of long-term care facility	38 (22)	34 (19)	.60
Outpatient GI/GU procedure	11 (6.2)	7 (4)	.33
Urinary catheter present	71 (40)	32 (18)	< .001
Central venous catheter	86 (49)	48 (27)	< .001
History of ESBLE	46 (26)	5 (2.8)	< .001
Hospital-acquired infection	77 (44)	49 (28)	.002
Hospitalization in previous 90 days	95 (54)	78 (44)	.07
Fluoroquinolone in previous 90 days	59 (33)	25 (14)	< .001
Beta-lactam in previous 90 days	90 (51)	63 (36)	.004
Cephalosporin in previous 90 days	90 (51)	45 (25)	< .001

In the univariate analysis, risk factors that achieved a significant difference included cirrhosis, AKI, presence of urinary catheter, presence of center venous catheter, history of an ESBLE infection, hospital-acquired infection, and recent fluoroquinolone, cephalosporin, or beta-lactam use (Table 1). Cirrhosis and AKI were present in 16% and 53% of ESBLE patients compared to 8% and 41% in the non-ESBLE group (*P* = .02 and *P* = .03, respectively). Presence of a urinary (40% vs 18%; *P* < .001) and/or a central venous catheter (49% vs 27%; *P* < .001) was more common in the ESBLE group compared to the non-ESBLE group. Those with a history of an ESBLE organism as well as patients with a hospital-acquired infection were more likely to be in the ESBLE group (*P* < .001 and *P* = .002, respectively). LOS was longer in the ESBLE group at 12 days compared to 7 days in the non-ESBLE group (*P* < 0.001).

The multivariate analysis showed that four factors were independently associated with an ESBLE infection: cirrhosis, urinary catheter, central venous catheter, and history of ESBLE (Table 2). Having a history of an ESBLE had the highest adjusted odds ratio (aOR 12.49; 95% CI 4.71–33.15, *P* < .001) of the four factors. Cirrhosis (aOR 2.28; 95% CI 1.10–4.76, *P* = .028), presence of a urinary catheter (aOR 2.73; 95% CI 1.62–4.62, *P* < .001), and presence of a central venous catheter (aOR 2.19; 95% CI 1.40–3.56, *P* = .002) had comparable adjusted odds ratios and were all statistically significant.

**Table 2.** Factors independently associated with ESBLE

	OR (95%CI)	aOR (95%CI)	*P*
Cirrhosis	2.19 (1.11–4.31)	2.28 (1.10–4.76)	.028
Urinary catheter	3.04 (1.87–4.94)	2.73 (1.62–4.62)	< .001
Central venous catheter	2.54 (1.63–3.96)	2.19 (1.40–3.56)	.002
History of ESBLE	12.08 (4.67–31.25)	12.49 (4.71–33.15)	< .001

Note. aOR, adjusted odds ratio; CI, confidence interval; OR, odds ratio.

## Discussion

This study retrospectively evaluated 354 individuals treated for infections at our institution and identified four factors that were independently associated with ESBLE (ie, cirrhosis, urinary catheter, central venous catheter, and history of ESBLE). Our study adds to a growing body of literature reporting risk factors for ESBLE infections. However, the lack of consistency in population, type of infection, and evaluated risk factors in previous studies make drawing conclusions difficult.

One of the largest variations in the literature of assessing ESBLE risk factors is the site of infection. For instance, some studies only evaluate risk factors in bloodstream infections, whereas others only evaluate risk factors in urinary tract infections (UTIs).^7,8,10–12^ While site-specific risk factor data are valuable, use may be limited as it cannot be generalized to other infection types or when the site of infection is unknown at the time of treatment initiation. In contrast, there are two published studies that evaluated all infection types, which may be more generalizable.^3,11^ We hypothesized evaluating all non-urine ESBLE-producing isolates would allow us to capture a generalizable sample, while not including an overly heterogenous population. Future studies are needed to evaluate whether all sites of infection should be included in one clinical decision support (CDS) tool or if different sites of infections should have their own unique CDS tools.

Another challenge to overcome when trying to use these findings in practice is the variation of risk factors evaluated throughout the literature; this includes both the list of potential risk factors collected as well as the definition of risk factors. For example, one study only analyzed ESBLE risk factors in UTIs and therefore evaluated risk factors relevant to that disease state not captured in previous studies, including neurogenic bladder and recurrent UTIs.^12^ Another example is the use of the Charlson comorbidity index, which was analyzed as a potential risk factor in three previous studies.^3,10,12^ One of those studies found that a score > 4 was independently associated with an ESBLE infection (aOR 3.8 [1.9–7.59]), whereas this was not found in the other two studies (aOR 0.83 [0.43–1.63]) and *P* = .507).^3,10,12^ Additionally, although most studies evaluated “prior antibiotic use” and the “duration of prior antibiotic use,” the definitions of these risk factors differed greatly among studies. Two studies analyzed antibiotics given 3 months preceding hospital admission,^3,12^ whereas another study analyzed antibiotics given 3 months preceding culture collection.^7^ With these differences in mind, we opted to collect antibiotic data 3 months prior to the culture, as this was most consistent with most studies. Duration of prior antibiotic use is also defined differently throughout the literature. Some studies only included antibiotics if therapy was given for at least 24 hours.^7,12^ This ranged from > 48 hours to > 72 hours in other studies.^3,10^ In contrast, our study evaluated antibiotic use regardless of duration. These variations highlight the importance of clear definitions and the need for appropriate education on an institution’s specific risk factors before rolling out an ESBLE risk score.

Our study showed that cirrhosis, presence of a urinary catheter, presence of a central venous catheter, and history of an ESBLE infection were independently associated with an ESBLE in the multivariate analysis. Previous studies also demonstrate that presence of a catheter may be a risk factor for ESBLE infections.^3,7,12^ The adjusted odds ratio for presence of urinary catheter in this study was 2.73 [1.62–4.62], which was very similar to that of the previous literature which ranged from 2.36 to 3.52.^3,7,12^ This finding has promising clinical implications as all four studies were conducted in vastly different populations; due to this finding, we believe that this factor should be evaluated by all institutions who are looking to develop an ESBLE risk score tool. Having a history of an ESBLE infection is another factor that both our study and multiple other studies identified an independent risk factor.^7,8,11^ While we acknowledge the adjusted odds ratios vary among studies (range 12.49 to 51.45), this was consistently the strongest predictor of ESBLE and should be included in future risk factor identification studies.^7,8,11^While both presence of a urinary catheter and history of an ESBLE seem to be the most promising risk factors, more studies are still needed describing implementation and evaluation of these risk factors in a CDS tool.

Limitations from our analysis include its retrospective nature and the dependence on previously documented data. We were not able to capture all variables that impact ESBLE risk due to this limitation. For example, travel history is not frequently documented in our electronic health record; thus, we were not able to capture it in our study. It is also possible that unidentified errors were made as data were originally entered into patient charts. Additionally, our study is limited in the number of total patients. Over the course of the study period, only 177 ESBLE infections met inclusion criteria, therefore only 177 matched non-ESBLE patients were included. This small sample size may have impacted the precision of our results. For instance, the confidence interval for the factor prior ESBLE infection spanned from 4.71 to 33.15. However, this is consistent with findings from previous studies like, Augustine et al. who reported an aOR of 26.8 and 95% CI, 7.0–108.2 for prior infections or colonization with ESBLE.^7^ We also acknowledge that during this study period, there were likely changes in the epidemiology of Gram-negative resistance both in the United States and worldwide. Furthermore, our study was not able to assess the difference between “colonization” with an organism versus “infection” with an organism. We tried to account for this by only including patients who received an antibiotic in hopes that the clinicians’ decisions to treat the organism helped represent a true infection. Another limitation is that we did not collect all social determinants of health; this should be noted when applying to different patient populations. Finally, the majority of our cultures were from the blood. Therefore, cultures from other sites were most likely not fairly represented in our analysis. Our study adds to the variable literature describing variable risk factors for Gram-negative infections. A future study validating previously published risk factors combined with our data will hope to offer a comprehensive CDS tool.

## Conclusion

This study identified four factors that were independently associated with an ESBLE infection in our patient population: cirrhosis, urinary catheter, central venous catheter, and history of ESBLE. Both presence of a catheter and history of an ESBLE infection have been demonstrated in previous studies. Future directions include externally validating the factors identified in our study to incorporate them in a risk score to be used as a CDS tool and/or validating a previously created risk score using our hospital data.
